# Supplementary material for: Physical Activity during Preconception Impacts Some Maternal Outcomes—A Cross-Sectional Study on a Population of Polish Women
Source: Int J Environ Res Public Health. 2023 Feb 17;20(4):3581. doi: 10.3390/ijerph20043581 (PMC9962747; doi:10.3390/ijerph20043581)
Supplement: Supplementary file 1 [file ijerph-20-03581-s001.zip › ijerph-2106624-supplementary.pdf]

Supplement S1. The questionnaire

1. How old were you during your last pregnancy?
    a. 16-20
    b. 21-25
    c. 26-30
    d. 31-35
    e. 36 or more
2. Where do you live?
    a. in the countryside
    b. in a town < 50,000 inhabitants
    c. in a city 50,000-100,000 inhabitants
    d. in a city 100,000-500,000 inhabitants
    e. in a city > 500.000 inhabitants
3. What is your education?
    a. primary
    b. vocational
    c. secondary
    d. higher
4. How many times have you been pregnant?
    a. once
    b. twice
    c. three times
    d. four times or more
5. Was your last pregnancy single or multiple?
    a. singleton pregnancy
    b. multiple pregnancy
6. How tall in centimeters are you?
7. How much did you weight before pregnancy?
8. How many kilograms did you gain during pregnancy?
9. Did you receive advice during your pregnancy from a qualified healthcare professional (doctor /midwife) about optimal pregnancy-related weight gain?
    a. yes
    b. no
10. Did you receive advice during your pregnancy from a qualified healthcare professional (doctor /midwife) regarding the recommended physical activity?
    a. yes
    b. no
11. Did a qualified healthcare professional (doctor/midwife) due to existing contraindications forbid physical activity during pregnancy?
    a. yes
    b. no
12. Did you have any of the following diseases/conditions during pregnancy?
    a. I had no contraindications for physical activity during pregnancy
    b. haemodynamically significant heart disease
    c. restrictive lung disease
    d. incompetent cervix/cerclage
    e. multiple gestation at risk for premature labour
    f. persistent second or third trimester bleeding
    g. placenta praevia after 26 weeks of gestation
    h. premature labour during the current pregnancy
    i. ruptured membranes

    j. pregnancy-induced hypertension
    k. other
13. Have you had gestational diabetes mellitus?
    a. yes
    b. no
    c. I had diabetes mellitus before pregnancy
14. How often did you exercise in the 6 months before pregnancy? Please rate your average activity during a week.
    a. less than 30 minutes per week
    b. 2-3 times for 30 minutes per week
    c. 4-5 times for 30 minutes per week
    d. more than 5 times for 30 minutes per week
15. If in the previous question you selected less than 30 minutes per a week, please state the reason for the low activity
16. How often did you exercise in the first trimester of pregnancy? Please rate your average activity during a week.
    a. less than 30 minutes per week
    b. 2-3 times for 30 minutes per week
    c. 4-5 times for 30 minutes per week
    d. more than 5 times for 30 minutes per week
17. What were the types of physical activity practiced by you in the first trimester of pregnancy? (multiple choice)
    a. walk
    b. march
    c. home gymnastics
    d. yoga
    e. fitness training
    f. swimming
    g. cycling
    h. horse riding
    i. full body workout
    j. contact sports
    k. other
18. How often did you exercise in the second trimester of pregnancy? Please rate your average activity during a week.
    a. less than 30 minutes per week
    b. 2-3 times for 30 minutes per week
    c. 4-5 times for 30 minutes per week
    d. more than 5 times for 30 minutes per week
19. What were the types of physical activity practiced by you in the second trimester of pregnancy? (multiple choice)
    a. walk
    b. march
    c. home gymnastics
    d. yoga
    e. fitness training
    f. swimming
    g. cycling
    h. horse riding
    i. full body workout
    j. contact sports

    k. other
20. How often did you exercise in the third trimester of pregnancy? Please rate your average activity during a week.
    a. less than 30 minutes per week
    b. 2-3 times for 30 minutes per week
    c. 4-5 times for 30 minutes per week
    d. more than 5 times for 30 minutes per week
21. What were the types of physical activity practiced by you in the third trimester of pregnancy? (multiple choice)
    a. walk
    b. march
    c. home gymnastics
    d. yoga
    e. fitness training
    f. swimming
    g. cycling
    h. horse riding
    i. full body workout
    j. contact sports
    k. other
22. What was the week of pregnancy at delivery?
23. How did you deliver your child?
    a. natural birth
    b. forceps delivery/delivery using a vacuum extractor
    c. caesarean section
24. If you selected a cesarean section in the previous question, please state the reason for the caesarean section
25. What was the newborn birth weight?
    a. <1500 g
    b. 1500-1999 g
    c. 2000-2499 g
    d. 2500-2999 g
    e. 3000-3499 g
    f. 3500-4000 g
    g. >4000 g
26. What was the newborn's birth length? (in centimeters)
